# *Evaluation of bleach-sedimentation for sterilising and concentrating *Mycobacterium tuberculosis *in sputum specimens*

**DOI:** 10.1186/1471-2334-11-269

**Published:** 2011-10-11

**Authors:** Rusheng Chew, Carmen Calderón, Samuel G Schumacher, Jonathan M Sherman, Luz Caviedes, Patricia Fuentes, Jorge Coronel, Teresa Valencia, Beatriz Hererra, Mirko Zimic, Lucy Huaroto, Ivan Sabogal, A Rod Escombe, Robert H Gilman, Carlton A Evans

**Affiliations:** 1 IFHAD: Innovation For Health And Development, London, UK; 2 University of Cambridge School of Clinical Medicine, Cambridge, UK; 3 Department of Microbiology, Faculty of Sciences and Philosophy, Universidad Peruana Cayetano Heredia, Lima, Peru; 4 Asociación Benefica Prisma, Lima, Peru; 5 Mayo Medical School, Rochester, Minnesota, USA; 6 Laboratory of Bioinformatics, Department of Biochemistry and Molecular Biology, Faculty of Sciences and Philosophy, Universidad Peruana Cayetano Heredia, Lima, Peru; 7 Department of Microbiology, Hospital Dos de Mayo, Lima, Peru; 8 Department of Microbiology, Hospital Daniel Carrión, Lima, Peru; 9 Department of Infectious Diseases and Immunity and Wellcome Centre for Clinical Tropical Medicine, Imperial College London, London, UK; 10 Johns Hopkins Bloomberg School of Public Health, Baltimore, Maryland, USA

## Abstract

**Background:**

Bleach-sedimentation may improve microscopy for diagnosing tuberculosis by sterilising sputum and concentrating *Mycobacterium tuberculosis*. We studied gravity bleach-sedimentation effects on safety, sensitivity, speed and reliability of smear-microscopy.

**Methods:**

This blinded, controlled study used sputum specimens (n = 72) from tuberculosis patients. Bleach concentrations and exposure times required to sterilise sputum (n = 31) were determined. In the light of these results, the performance of 5 gravity bleach-sedimentation techniques that sterilise sputum specimens (n = 16) were compared. The best-performing of these bleach-sedimentation techniques involved adding 1 volume of 5% bleach to 1 volume of sputum, shaking for 10-minutes, diluting in 8 volumes distilled water and sedimenting overnight before microscopy. This technique was further evaluated by comparing numbers of visible acid-fast bacilli, slide-reading speed and reliability for triplicate smears before versus after bleach-sedimentation of sputum specimens (n = 25). Triplicate smears were made to increase precision and were stained using the Ziehl-Neelsen method.

**Results:**

*M. tuberculosis *in sputum was successfully sterilised by adding equal volumes of 15% bleach for 1-minute, 6% for 5-minutes or 3% for 20-minutes. Bleach-sedimentation significantly decreased the number of acid-fast bacilli visualised compared with conventional smears (geometric mean of acid-fast bacilli per 100 microscopy fields 166, 95%CI 68-406, versus 346, 95%CI 139-862, respectively; *p *= 0.02). Bleach-sedimentation diluted paucibacillary specimens less than specimens with higher concentrations of visible acid-fast bacilli (*p *= 0.02). Smears made from bleach-sedimented sputum were read more rapidly than conventional smears (9.6 versus 11.2 minutes, respectively, *p *= 0.03). Counting conventional acid-fast bacilli had high reliability (inter-observer agreement, r = 0.991) that was significantly reduced (*p *= 0.03) by bleach-sedimentation (to r = 0.707) because occasional strongly positive bleach-sedimented smears were misread as negative.

**Conclusions:**

Gravity bleach-sedimentation improved laboratory safety by sterilising sputum but decreased the concentration of acid-fast bacilli visible on microscopy, especially for sputum specimens containing high concentrations of *M. tuberculosis*. Bleach-sedimentation allowed examination of more of each specimen in the time available but decreased the inter-observer reliability with which slides were read. Thus bleach-sedimentation effects vary depending upon specimen characteristics and whether microscopy was done for a specified time, or until a specified number of microscopy fields had been read. These findings provide an explanation for the contradictory results of previous studies.

## Background

Sputum smear-microscopy is the most widely used laboratory test for diagnosing tuberculosis but in poorly equipped settings can expose laboratory staff to the infectious pathogen *Mycobacterium tuberculosis *[1]. Consequently, the risk of tuberculosis disease has been found to be 7-79 times greater in laboratory staff than the general population [2]. Bleach is bactericidal and adding bleach to sputum may sterilise it, potentially protecting staff from tuberculosis infection during processing although this would also prevent subsequent culture based testing. However, the sterilising activity of bleach is poorly characterised for *M. tuberculosis *and the bleach concentrations and exposure times required during bleach-sedimentation to sterilise sputum and prevent biohazard to staff are unknown [3-6].

Smear-microscopy fails to diagnose patients who have low concentrations of *M. tuberculosis *in their sputum, hampering tuberculosis control. Conventional smear-microscopy involves smearing sputum on a microscope slide that is then stained and examined by high power microscopy to detect the causative acid-fast bacillus *M, tuberculosis*. For a 50% probability of finding a single acid-fast bacillus in 100 microscopy fields, approximately 5, 000 acid-fast bacilli must be present per ml of sputum [7]. Consequently the sensitivity of this technique is typically only 30-70% of the sensitivity of culture [8,9]. Tuberculosis patients who have AIDS and/or are children usually have lower concentrations of *M, tuberculosis *bacilli in their sputum, so the diagnostic sensitivity of smear-microscopy is lower in these patients [10-12]. Thus, reliance on smear-microscopy may cause missed or delayed tuberculosis diagnosis, potentially increasing morbidity, mortality and tuberculosis transmission. Increasing the sensitivity of tuberculosis diagnostic testing is a public health priority.

Diagnostic sensitivity increases if acid-fast bacilli are concentrated into the small volume that can be visualised by microscopy. Bleach-sedimentation has been hypothesised to concentrate acid-fast bacilli in sputum specimens and in support of this hypothesis a recent meta-analysis reported that bleach-sedimentation caused a 9% increase in tuberculosis diagnostic sensitivity compared to conventional smear-microscopy [13]. Centrifugation concentrates *M. tuberculosis *and is used in some bleach-sedimentation protocols but centrifuges are expensive, may create biohazardous aerosols and are infrequently available in resource-poor settings. We therefore restricted our research to gravity bleach-sedimentation techniques that do not involve centrifugation [14-18].

Most studies of bleach-sedimentation reported that it slightly increased diagnostic sensitivity of smear-microscopy [6,19]. Variations between these studies may be explained by failure to record the number of microscopy fields examined and/or time spent performing microscopy and by difficulty making blinded comparisons because bleach-sedimentation changes the appearance of sputum smears [19]. There were also differences in protocol: 5 published bleach-sedimentation techniques share a common initial step of mixing sputum with an equal volume of 5% bleach, which is then either stained without further dilution [17,18] or after dilution in water [14-16]. Dilution in water after adding bleach may reduce bleach-mediated damage to *M. tuberculosis *that can inhibit subsequent acid-fast staining [6]. All bleach-sedimentation techniques involve some dilution of sputum and it is unknown whether they cause overall concentration or dilution of visible acid-fast bacilli [6,19].

Most microscopy studies have compared either rates of microscopy positivity or alternatively the numbers of slides in each categorical microscopy grade (negative, weakly positive '+', positive '++', or strongly positive '+++'; see figure legends for definitions). These approaches are clinically relevant but are insensitive for assessing bleach-sedimentation because few specimens contain concentrations of acid-fast bacilli close to the threshold between microscopy grades. Consequently, when this categorical approach is used large numbers of specimens must be studied and small effects of bleach-sedimentation may be missed. The use of a more precise assessment of acid-fast bacilli concentration such as the number visible per 100 high-powered microscopy fields should facilitate characterisation of bleach-sedimentation effects.

Bleach-sedimentation lyses human cells within sputum, which clears the field of view during microscopy and may accelerate slide reading speed but these effects do not appear to have been quantified [6] and confound assessment of acid-fast bacilli concentrations. Consequently, it is unclear from published research whether bleach-sedimentation increases the concentration of visible acid-fast bacilli, increases the amount of sputum examined in the available time, neither or both of these effects. To overcome these limitations we developed a protocol using triplicate slides from each specimen before and after bleach-sedimentation to characterise effects on smear-microscopy for each specimen.

We used these methodological refinements to characterise the effect of bleach-sedimentation on the safety, sensitivity, speed and reliability of smear-microscopy. This novel methodology clarifies the specific effects of bleach-sedimentation and provides an explanation for the discrepant results from previous studies.

## Methods

### Study design

**Figure 1:**
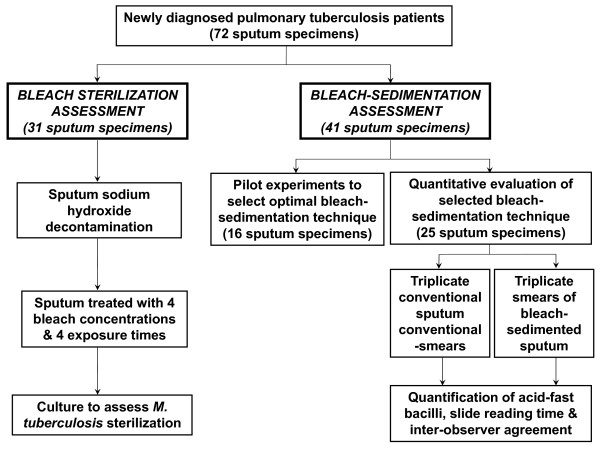
**Study flowchart**. The Standards for the Reporting of Diagnostic Accuracy (STARD) flowchart for the study. Results were available for all of the procedures planned in the study protocol.

Figure 1 shows the study design that involved 72 sputum specimens. First, bleach-sterilisation studies determined the bleach exposure required to increase laboratory safety by sterilising sputum. In the light of these results, 5 bleach-sedimentation protocols that would completely sterilise sputum were compared in pilot experiments. The best performing of these techniques was then assessed in detail. Sample size calculations were not performed because the concentration of acid-fast bacilli in specimens for the planned protocol was unknown. All experimentation was performed blinded to the results of all other tests, at room temperature, and all slides were read in random order.

### Setting

The study was carried out over a 6-month period in shantytowns in Lima, Peru in which tuberculosis principally affects socioeconomically disadvantaged people [20]. Peru is a middle-income country with high tuberculosis incidence in which conventional Ziehl-Neelsen sputum smear-microscopy is the principal diagnostic test for tuberculosis.

### Specimens

In collaboration with the national tuberculosis control program, sputum specimens were obtained on the day that they had been found by local laboratories to be microscopy-positive for acid-fast bacilli. All specimens were from untreated, newly diagnosed patients being investigated for clinically suspected tuberculosis. We recorded the volume and consistency (whether salivary or mucoid) of each specimen and whether the microscopist was moderately or very experienced.

#### (1) Bleach sterilisation assessment

Assessment of bleach-sterilisation utilised 31 sputum specimens that were homogenised and decontaminated with the sodium hydroxide N-acetyl cysteine method [3]. Briefly, a freshly prepared solution of 4% sodium hydroxide, 2.9% sodium citrate and 0.5% N-acetyl cysteine (Sigma, Saint Louis, Missouri) was mixed with an equal volume of sputum and left for 15 minutes. The decontamination was then stopped by adding a 7-times excess volume of phosphate-buffered saline (PBS, pH 6.8), centrifuging at 3, 000 × *g *for 20 minutes and discarding the supernatant. The addition of a 7-times excess volume of PBS and the centrifugation conditions are standard practices for centrifuge-decontamination in some laboratories in Peru because these conditions were found in pilot experiments to provide optimal neutralisation and concentration (data not shown). The pellet from centrifugation was re-suspended in 34 ml PBS and then split into 17 aliquots that were each 2 ml in volume. One aliquot was used as a control to which no bleach was added and 2 ml of 3%, 6%, 10% and 15% bleach were each added to quadruplet sets of each of the other aliquots. The bleach dilutions were prepared fresh from commercially available 15% bleach (sodium hypochlorite, NaOCl; Import Export Lider, Lima, Peru). Each of the bleach-sputum mixtures was treated with bleach for 1, 5, 10 or 20 minutes. After this exposure to a total of 16 combinations of bleach concentrations and exposure times, reactions were stopped by adding a 7-times excess volume of PBS and shaking by hand until homogenised. The solution was then centrifuged at 3,000 × *g *for 15 minutes, the supernatant was discarded and the pellet re-suspended in 0.2% bovine serum albumin (Sigma, Saint Louis, Missouri). The entire re-suspended pellet was then spread on a Middlebrook 7H11 agar plate (Difco, Detroit, Michigan) supplemented with 10% oleic acid, albumin, dextrose and catalase as described [3]. The plate was sealed in a Ziploc^® ^bag (Johnson, Wisconsin) to prevent drying, incubated at 37°C in air and inspected for *M. tuberculosis *growth using an inverted microscope twice weekly for 8 weeks.

#### (2) Bleach-sedimentation assessment

##### Pilot work for selection of bleach-sedimentation technique

In pilot experiments, 5 published bleach-sedimentation protocols [14-18] were compared to select an optimal technique for further assessment. Triplicate conventional smears were prepared from 16 sputum specimens. The remainder of each specimen was then processed by these 5 protocols after which triplicate slides were produced from each bleach-sedimented specimen. Bleach-sedimentation without subsequent water dilution followed by sedimentation for 30-45 minutes [18] or 12-15 hours [17] considerably reduced the number of acid-fast bacilli visible on microscopy, possibly through bleach damaging *M. tuberculosis *(data not shown), so these techniques were not further assessed. The other techniques involved adding bleach to the sputum without shaking [14], shaking at regular intervals for 15 minutes [16] or continuous shaking for 10 minutes [15] before dilution with water followed by sedimentation. These 3 techniques had similar effects on the numbers of acid-fast bacilli visible on microscopy (data not shown). The last of these 3 techniques [15] had the most precisely defined methodology and was reported to have produced optimal results so was selected for the further evaluation described below.

##### Quantitative evaluation of bleach-sedimentation

The bleach-sedimentation method described by Gebre-Selassie [15] selected in our pilot work was further assessed as follows. Triplicate conventional smears were prepared from each of 25 sputum specimens to serve as controls. The remaining volume of each specimen up to a maximum volume of 1.5 ml was then placed into 15 ml polypropylene tubes (Falcon BD, San Jose, California). Fresh 5% bleach was prepared by dilution from a solution of 8% bleach that the manufacturer reported contained 8.09 g/100 ml free chlorine ions and had 1.125 g/ml density. One volume of 5% bleach (equal to the sputum volume) was added to each specimen and the mixture was shaken by hand continuously for 10 minutes. Eight volumes of distilled water were then added and the mixture was left to sediment for 16 hours. The supernatant was then pipetted off and the pellet, or the basal approximately 250 μl if no pellet had formed, was mixed by pipetting and used to prepare triplicate smears.

##### Smear preparation

In order to standardise the amount of sputum applied to all slides, a pipette was used to apply to each slide 40 μl of unprocessed or bleach-sedimented sputum that was smeared over a single area of approximately 1 cm × 2 cm. Forty μl was used because this volume was equivalent to 1 drop of sputum. Slides were air-dried, heat-fixed by passing over a flame and stained using the Ziehl-Neelsen method. Briefly, the smear was flooded with 0.3% carbol fuchsin, heated with a flame, left to stand for 10 minutes and washed with water. Acid-alcohol was applied for 2 minutes, the slide was washed with water and the counter-stain methylene blue was applied for 1 minute, washed off and slides left to dry vertically [3].

##### Smear-microscopy sensitivity

The number of acid-fast bacilli was counted in 100 high-power fields that were read per slide using standard oil-immersion light microscopy. If < 32 acid-fast bacilli were visible in 100 fields then an additional 200 fields were read. This cut-off was derived because it is the mid-point between 10 and 100 on a logarithmic scale and was selected arbitrarily to increase the precision of quantification of relatively low concentrations of acid-fast bacilli.

##### Smear-microscopy speed

Microscopists recorded the time they spent counting the acid-fast bacilli per 100 fields on each slide. To improve the blinded nature of the comparison, they were unaware of our research hypotheses and recorded the time spent on each slide as a laboratory routine.

##### Smear-microscopy reliability

Both microscopists cross read a random sample of 1 in 8 slides to determine the degree of agreement between their readings using the same protocol as the first slide reading.

##### Ethical considerations

Ethical committee approval was not required because this research did not involve human subjects or patient diagnosis and utilised anonymized, unlinked excess specimens that would otherwise have been discarded.

##### Statistical analysis

Data were analysed using SPSS 11.5 software (SPSS Corp., Chicago, Illinois). Acid-fast bacilli count data were non-Gaussian and were summarised using geometric means with 95% confidence intervals (95%CI) and were compared with the non-parametric Wilcoxon signed-rank test (for paired data) and the rank sum test (for unpaired data). Slide-reading time data were Gaussian and were summarised using arithmetic means with standard errors of the mean (SEM) that were compared using the paired Student's *t*-test. Correlations were assessed with the non-parametric Spearman's rank correlation coefficient (r). All *p*-values corresponded to 2-sided hypothesis testing.

## Results

### (1) Bleach sterilisation assessment

**Figure 2:**
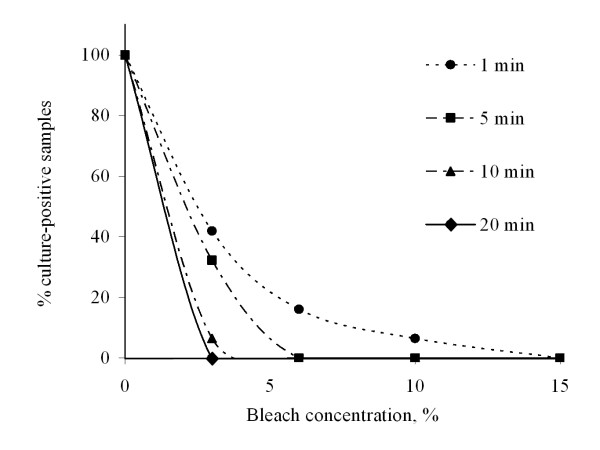
**Bleach sterilization**. *M. tuberculosis *viability after treatment of sputum specimens with 4 different bleach concentrations for 4 different exposure times, i.e. a total of 16 combinations of bleach concentrations and exposure times. All control specimens were culture-positive.

Figure 2 shows bleach effects on *M. tuberculosis *viability. All control specimens that had not been treated with bleach were culture-positive. The proportion of specimens sterilised increased with bleach concentration and exposure time. All specimens were sterilised by exposure to 15% bleach for 1 minute, 6% bleach for 5 minutes or 3% bleach for 20 minutes.

### (2) Bleach-sedimentation assessment

The median volume of the sputum specimens was 1.5 ml (inter-quartile range 1.0-7.0 ml) and 28% of specimens were classified as mucoid, not salivary.

### Smear-microscopy sensitivity

**Figure 3:**
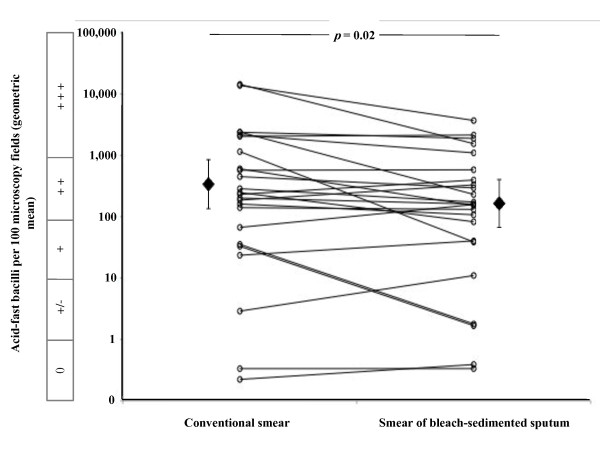
**Effect of bleach-sedimentation on the concentration of acid-fast bacilli**. The number of acid-fast bacilli visualised by smear-microscopy is shown. Each of the open circles represents the geometric mean number of acid-fast bacilli visible in 100 microscopy fields for triplicate, identically prepared slides. Each line joins the data derived from 1 of the 25 sputum specimens i.e. the geometric mean of triplicate conventional smear-microscopy slides (the left-hand end of each line) versus the geometric mean of triplicate slides prepared after bleach-sedimentation (the right-hand end of each line). The filled diamonds represent the geometric mean of all 25 specimens and the error bars represent 95% confidence intervals. The box parallel with the vertical axis indicates the smear-microscopy grade equivalent to the acid-fast bacilli counts per 100 microscopy fields (0 indicates none visible/100 fields; +/- indicates 1-9/100 fields; + indicates 10-99/100 fields; ++ indicates 100-999/100 fields; and +++ indicates > 1, 000/100 fields).

In Figure 3, the circles represent geometric mean numbers of acid-fast bacilli visible in triplicate smears and each line joins the data derived from 1 specimen. Bleach-sedimentation significantly reduced the number of acid-fast bacilli visible. Specifically, the geometric mean number of acid-fast bacilli visible fell significantly from 346 for all conventional smears to 166 for all smears prepared from the same specimens after bleach-sedimentation (Table 1; *p *= 0.02). Bleach-sedimentation involved a 10-fold dilution of specimens and the decrease in the number of acid-fast bacilli visible was significantly less than 10-fold (*p *= 0.001).

**Table 1:** Comparison of conventional smears and smears prepared from bleach-sedimented sputum

	Conventional smears	Smears prepared from bleach-sedimented sputum *	*p*-value
Acid-fast bacilli counts per 100 microscopy fields, geometric mean (95% CI)	346 (139-862)	166 (68-406)	0.02
Slide-reading time, arithmetic mean minutes (standard error of the mean)	11.2 (0.92)	9.6 (0.69)	0.03
Inter-observer agreement, correlation coefficient (r)	0.991	0.997	-
Correlation between concentrating effect of bleach-sedimentation and acid-fast bacilli counts, correlation coefficient (r)	-0.46		0.02
Correlation between concentrating effect of bleach-sedimentation and volume of the sputum specimen, correlation coefficient (r)	-0.13		0.7
Concentrating effect of bleach-sedimentation comparing salivary versus mucoid sputum specimens	-		0.3
Concentrating effect of bleach-sedimentation comparing moderately versus very experienced microscopists	-		0.6

* False-negative (zero) microscopy readings by a single microscopist for 3 strongly positive smears prepared from bleach-sedimented specimens were excluded when calculating these data (see text and Figure 5).

**Figure 4:**
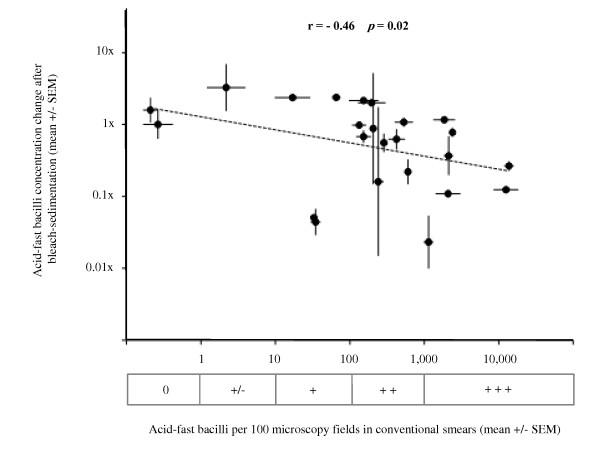
**Correlation between the numbers of acid-fast bacilli visible by sputum smear-microscopy and the bleach-sedimentation concentrating effect**. Each data point represents data from a single specimen. The horizontal axis shows the geometric mean number of acid-fast bacilli per 100 microscopy fields visualised in triplicate conventional smears. The horizontal error bars represent the standard error of the mean (SEM) for these triplicate data. The vertical axis shows the mean change in the number of acid-fast bacilli per 100 microscopy fields visualised in triplicate slides prepared after bleach-sedimentation of that specimen. The vertical error bars represent the SEM for these triplicate data. The diagonal broken line is the regression line. r represents the correlation coefficient. The box parallel with the horizontal axis indicates the smear-microscopy grade equivalent to the acid-fast bacilli counts per 100 microscopy fields (0 indicates none visible/100 fields; +/- indicates 1-9/100 fields; + indicates 10-99/100 fields; ++ indicates 100-999/100 fields; and +++ indicates > 1000/100 fields).

To assess whether the effect of bleach-sedimentation varied with the concentration of acid-fast bacilli, the geometric mean number of acid-fast bacilli per 100 microscopy fields for triplicate conventional smears was compared with the change in the count of acid-fast bacilli for triplicate smears prepared after bleach-sedimentation of each specimen (Figure 4). Bleach-sedimentation reduced the counts of acid-fast bacilli significantly less in paucibacillary specimens containing fewer acid-fast bacilli than in specimens with higher concentrations of acid-fast bacilli (Table 1; r = -0.46; *p *= 0.02). Both before and also after bleach sedimentation, there was minimal variance in the counts of acid-fast bacilli from triplicate independently counted slides from each specimen, demonstrating that the phenomenon of regression to the mean could not explain the association that we observed between the effect of bleach-sedimentation and the concentration of acid-fast bacilli. There was no significant association between the effect of bleach-sedimentation on the counts of visible acid-fast bacilli and the sputum volume, sputum viscosity, nor experience of the microscopist (Table 1; all *p *> 0.1).

### Smear-microscopy speed

It took a mean of 11.2 minutes to read conventional smears versus 9.6 minutes for slides prepared from bleach-sedimented sputum (Table 1; *p *= 0.03). Therefore, bleach-sedimentation resulted in a mean 1.6-minute (14%) decrease in the time taken for microscopy.

### Smear-microscopy reliability

**Figure 5:**
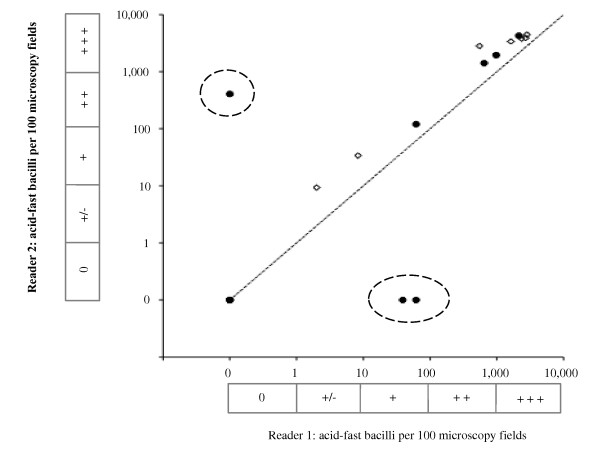
**Microscopist inter-observer agreement**. The inter-observer agreement is shown for 2 microscopists for conventional smears and for smears prepared after bleach-sedimentation. Filled circles represent smears prepared from bleach-sedimented sputum (correlation coefficient r = 0.707), and open diamonds represent conventional smears (r = 0.991). The 3 data points encircled by broken lines represent smears prepared from bleach-sedimented sputum found to be clearly positive by one reader but negative by the other reader. Excluding these 3 false-negative results caused the value of the correlation coefficient for results from bleach-sedimented sputum to increase to r = 0.997. The dotted line represents perfect agreement. The boxes parallel with the axes indicate the smear-microscopy grade equivalent to the acid-fast bacilli counts per 100 microscopy fields (0 indicates none visible/100 fields; +/- indicates 1-9/100 fields; + indicates 10-99/100 fields; ++ indicates 100-999/100 fields; and +++ indicates > 1000/100 fields).

Figure 5 shows the number of acid-fast bacilli in slides assessed by 2 microscopists. There was significant (*p *< 0.01) inter-observer agreement for all smears and for conventional smears inter-observer agreement was significantly (*p *= 0.003) higher (r = 0.991) than for smears prepared from bleach-sedimented sputum (r = 0.707). This significantly reduced level of agreement after bleach-sedimentation was entirely explained by discordant readings for 3 slides prepared from salivary (i.e. non-mucoid) specimens (encircled in Figure 5): reader 1 reported a slide as negative that reader 2 reported as strongly positive and reader 2 reported 2 slides as negative that reader 1 reported as strongly positive. The other slides made from these 3 specimens were reported as strongly positive by both microscopists. Excluding these 3 false-negative readings caused the inter-observer agreement for bleach-sedimented specimens to increase significantly (*p *< 0.001) to r = 0.997, higher than for conventional smears.

## Discussion

This study defined the bleach concentrations and exposure times needed to sterilise *M. tuberculosis *in sputum specimens and demonstrated that bleach-sedimentation sterilised sputum and increased microscopy reading speed. However, bleach-sedimentation decreased the concentration of visible acid-fast bacilli, especially in specimens with higher concentrations of acid-fast bacilli and caused occasional false-negative microscopy results.

Past studies of the sterilising activity of bleach on *M. tuberculosis *have incompletely defined the required concentrations and treatment times [6]. For example, Kent and Kubica [3] stated that 0.1-0.5% bleach was sufficient to sterilise *M. tuberculosis*, but the necessary exposure time was not reported. Best et al. [4] found that *M. tuberculosis *suspended in sputum required 1 minute of exposure to 1% bleach to reduce the number of colony-forming units. These results are consistent with our finding that increasing either the exposure time or the bleach concentration increased sterilisation. Our finding that bleach sterilises sputum at the concentrations and exposure times used for bleach-sedimentation demonstrates that this technique has the potential to improve biosafety in diagnostic laboratories. This may be important for basic laboratories in resource-poor settings that lack biosafety cabinets, masks and other facilities to reduce the biohazard associated with handling infectious sputum [1,2].

Studies evaluating bleach-sedimentation protocols have provided conflicting results [6,13-19]. We evaluated the bleach-sedimentation technique that previous studies and our pilot experiments suggested has greatest efficacy. We found that this bleach-sedimentation technique decreased the number of visible acid-fast bacilli and that this decrease was less than the 10-fold dilution involved in the protocol. Dilution with water after mixing sputum with bleach reduces *M. tuberculosis *exposure to high bleach concentrations, potentially preventing bleach from impairing acid-fast staining. However, our research demonstrated that this protocol caused an overall reduction in the number of visible acid-fast bacilli. Thus, published reports that bleach-sedimentation slightly increased diagnostic sensitivity may have resulted from bleach-sedimentation increasing microscopy speed and clarity rather than increased concentrations of acid-fast bacilli.

Bleach-sedimentation caused significantly less dilution of acid-fast bacilli in paucibacillary specimens than in specimens with higher concentrations of acid-fast bacilli. Although our quantification could have been affected by reader fatigue in counting high concentrations of acid-fast bacilli, this would not explain our finding that the concentrating effect of bleach-sedimentation varies according to the concentration of *M. tuberculosis *in the specimen. Since paucibacillary specimens occur with different frequency in different settings, this finding potentially explains the inconsistent results of past research.

Previous studies have reported subjective impressions of improved slide-reading efficiency with bleach treatment [15,16,21]. Bleach-processing required additional technician time to dilute the bleach, add it to each specimen, shake each specimen for 10 minutes and then dilute the specimen in water. The subsequent 16-hour overnight sedimentation period delayed microscopy, but did not require additional technician time. We found that bleach-sedimentation resulted in more rapid slide reading compared to conventional smear-microscopy. This decrease in slide-reading time after bleach-sedimentation may be explained by the digestive cell-lysing properties of bleach causing clearer microscopy fields that are free from human cells. This may facilitate identification of acid-fast bacilli because human cells may obscure acid-fast bacilli in conventional smears made from untreated sputum. A drawback of the protocol that we used is the requirement for the specimen to be left overnight, delaying results. However, throughput may not be delayed in laboratories that process specimens in batches.

The assessment of agreement between the 2 microscopists revealed discrepant readings of slides made from 3 strongly positive specimens. Bleach-sedimentation of salivary sputum causes the stained area of the slide to be invisible to the naked eye, probably allowing the microscopist to have accidentally examined the wrong area of the slide in these 3 cases. This would be expected to occur most frequently in high-throughput conditions. This should be preventable by marking the smear area on the underside of the slide before staining, which we recommend for future work. Damage of *M. tuberculosis *or washing of the sputum off the slide due to bleach exposure are unlikely alternative explanations because in each case another reader reported the same slide to be strongly positive.

One objective of bleach-sedimentation is to increase the sensitivity of smear-microscopy sufficiently to visualise acid-fast bacilli in paucibacillary specimens in which no acid-fast bacilli are visible on conventional smears and a limitation of our study was the use of only microscopy-positive specimens. Another limitation was the need for specimen decontamination in the sterilisation study that may have led to an overestimation of the sterilising potency of bleach. The use of antibiotic-enriched culture media selective for *M. tuberculosis *may overcome this problem by allowing the specimens to be cultured without decontamination [22,23]. Future work may optimise bleach-sedimentation by modifying bleach concentrations, sedimentation times, improving bleach neutralisation, by processing larger sputum volumes or by using filtration [24]. It would be useful to assess the effect of bleach-sedimentation on fluorescence microscopy [25], to measure the free chlorine content of bleach at the point of use rather than utilising the manufacturer's data and to characterise the effect of bleach storage [4,6].

## Conclusions

This novel methodology generated sensitivity and microscopy-reading speed data from each specimen by comparing triplicate conventional smears versus triplicate smears prepared after bleach-sedimentation. The results appear to explain the contradictory findings of previous studies by demonstrating that bleach-sedimentation reduced the concentration of acid-fast bacilli visible on microscopy, that this effect increased with the concentration of acid-fast bacilli in the specimen, that bleach-sedimentation allowed more of the specimen to be examined in the time available and caused occasional false-negative results.

Therefore, the effect of bleach-sedimentation will vary with 3 factors: the concentration of acid-fast bacilli in the specimen, whether slide reading involves examining a defined number of microscopy fields or examining slides for a defined period of time and the care with which each slide is examined. Differences in these variables will cause heterogeneous findings from evaluations of bleach-sedimentation in different settings. Specifically, our results suggest that evaluation of bleach-sedimentation in a setting with mainly paucibacillary specimens in which the duration of examination of each slide is fixed and performed carefully is more likely to demonstrate advantageous bleach-sedimentation effects than evaluation in a setting with few paucibacillary specimens, where a fixed number of microscopy fields are examined for each specimen in high-throughput conditions.

Thus, our findings validate a new methodology for evaluating techniques that aim to increase the sensitivity of sputum smear-microscopy and clarify the results of previous operational assessments of bleach-sedimentation by providing an explanation for their discrepant findings.

## List of abbreviations

CI: confidence interval; PBS: phosphate-buffered saline; SEM: standard error of the mean; ml: millilitre; AIDS: Acquired Immune Deficiency Syndrome; *M. tuberculosis: Mycobacterium tuberculosis*; cm: centimetres; μl: micro litres.

## Competing interests

The authors declare that they have no competing interests.

## Authors' contributions

RC and JMS led the bleach research except for the sterilisation work that was led by CC, with the supervision of RHG, LC, PF, TRV and BH. LH and IS provided clinical specimens and microbiological consultancy. MZ and SGS led data analysis. ARE provided editorial assistance. All authors contributed to manuscript preparation that was led by RC and SGS. The project was coordinated by CAE. All authors approved the manuscript.

## Pre-publication history

The pre-publication history for this paper can be accessed here:

http://www.biomedcentral.com/1471-2334/11/269/prepub

## References

[B1] Nyirenda TE Mundy CJ Harries AD Banerjee A Salaniponi FM Safety in laboratories carrying out sputum smear microscopy: a dilemma for resource-poor countries Int J Tuberc Lung Dis 1998 2 690 3 9712286

[B2] Joshi R Reingold AL Menzies D Pai M Tuberculosis among health-care workers in low- and middle-income countries: a systematic review PLoS Med 2006 3 e494 10.1371/journal.pmed.0030494 17194191 PMC1716189

[B3] Kent PT Kubica GP Public health mycobacteriology: A guide for the level III laboratory 1985 Atlanta: Centers for Disease Control

[B4] Best M Sattar SA Springthorpe VS Kennedy ME Efficacies of selected disinfectants against Mycobacterium tuberculosis J Clin Microbiol 1990 28 2234 2239 2121783 10.1128/jcm.28.10.2234-2239.1990 PMC268154

[B5] Ängeby KAK Alvarado-Galvez C Pineda-Garcia L Hoffner SE Improved microscopy for a more sensitive diagnosis of pulmonary tuberculosis Int J Tuberc Lung Dis 2000 4 684 687 10907772

[B6] Ängeby KAK Hoffner SE Diwan VK Should the 'bleach microscopy method' be recommended for improved case detection of tuberculosis? Literature review and key person analysis Int J Tuberc Lung Dis 2004 8 806 815 15260270

[B7] Toman K Tuberculosis: Case finding and Chemotherapy 1979 Geneva: World Health Organisation

[B8] Aber VR Allen BW Mitchison DA Ayuma P Edwards EA Keyes AB Laboratory studies on isolated positive cultures and the efficiency of direct smear examination Tubercle 1980 61 123 133 10.1016/0041-3879(80)90001-X 6777919

[B9] Wilkinson D Sturm AW Diagnosing tuberculosis in a resource-poor setting: the value of sputum concentration Trans R Soc Trop Med Hyg 1997 91 420 421 10.1016/S0035-9203(97)90263-7 9373638

[B10] Vijayakumar M Bhaskaram P Hemalatha P Malnutrition and childhood tuberculosis J Trop Pediatr 1990 36 294 298 2280436 10.1093/tropej/36.6.294

[B11] Bruchfeld J Aderaye G Palme IB Bjorvatn B Källenius G Lindquist L Sputum concentration improves diagnosis of tuberculosis in a setting with a high prevalence of HIV Trans R Soc Trop Med Hyg 2000 94 677 680 10.1016/S0035-9203(00)90230-X 11198655

[B12] Oberhelman R Soto G Gilman RH Caviedes L Castillo M Kolevic L Del Pino T Saito M Salazar E Negron E Laguna A Moore DA Evans CA New diagnostic approaches for pediatric TB among Peruvian children Lancet Infect Dis 2010 10 612 20 10.1016/S1473-3099(10)70141-9 20656559 PMC2975578

[B13] Cattamanchi A Davis JL Pai M Huang L Hopewell PC Steingart KR Does bleach-processing increase the accuracy of sputum smear microscopy for diagnosing pulmonary tuberculosis? J Clin Microbiol 2010 48 2433 2439 10.1128/JCM.00208-10 20421442 PMC2897477

[B14] Van Deun A Maug AK Cooreman E Hossain MA Chambuganj N Rema V Marandi H Kawria A Portaels F Bleach-sedimentation method for increased sensitivity of sputum smear microscopy: does it work? Int J Tuberc Lung Dis 2000 4 371 376 10777088

[B15] Gebre-Selassie S Evaluation of the concentration sputum smear technique for the laboratory diagnosis of pulmonary tuberculosis Trop Doct 2003 33 160 162 12870603 10.1177/004947550303300313

[B16] Miörner H Ganlöv G Yohannes Z Adane Y Improved sensitivity of direct microscopy for acid-fast bacilli: sedimentation as an alternative to centrifugation for concentration of tubercle bacilli J Clin Microbiol 1996 34 3206 3207 8940473 10.1128/jcm.34.12.3206-3207.1996 PMC229484

[B17] Farnia P Mohammadi F Zarifi Z Tabatabee DJ Ganavi J Ghazisaeedi K Farnia PK Gheydi M Bahadori M Masjedi MR Velayati AA Improving sensitivity of direct microscopy for detection of acid-fast bacilli in sputum: use of chitin in mucus digestion J Clin Microbiol 2002 40 508 511 10.1128/JCM.40.2.508-511.2002 11825964 PMC153416

[B18] Yassin MA Cuevas LE Gebrexabher H Squire SB Efficacy and safety of short-term bleach digestion of sputum in case-finding for pulmonary tuberculosis in Ethiopia Int J Tuberc Lung Dis 2003 7 678 683 12870690

[B19] Steingart KR Ng V Henry M Hopewell PC Ramsay A Cunningham J Urbanczik R Perkins MD Abdel Aziz M Pai M Sputum processing methods to improve the sensitivity of smear microscopy for tuberculosis: a systematic review Lancet Infect Dis 2006 6 664 674 10.1016/S1473-3099(06)70602-8 17008175

[B20] Rocha C Montoya R Zevallos K Curatola A Ynga W Franco J Fernandez F Becerra N Sabaduche M Tovar MA Ramos E Tapley A Allen NR Onifade DA Acosta CD Maritz M Concha DF Schumacher SG Evans CA The Innovative socioeconomic interventions against TB (ISIAT) project - an operational assessment Int J Tuberc Lung Dis 2011 15 s50 57 10.5588/ijtld.10.0447 21740659 PMC3160483

[B21] Habeenzu C Lubasi D Fleming AF Improved sensitivity of direct microscopy for detection of acid-fast bacilli in sputum in developing countries Trans R Soc Trop Med Hyg 1998 92 415 416 10.1016/S0035-9203(98)91071-9 9850395

[B22] Mitchison DA Allen BW Lambert RA Selective media in the isolation of tubercle bacilli from tissues J Clin Pathol 1973 26 250 252 10.1136/jcp.26.4.250 4349715 PMC477698

[B23] Grandjean L Martin L Gilman R Valencia T Herrera B Quino W Ramos E Rivero M Montoya R Escombe A Coleman D Mitchison D Evans CA TB & MDRTB testing by direct sputum culture in selective broth without decontamination or centrifugation J Clin Microbiol 2008 46 2339 44 10.1128/JCM.02476-07 18448689 PMC2446921

[B24] Ramos E Schumacher SG Siedner M Herrera B Quino W Alvarado J Montoya R Grandjean L Martin L Sherman JM Gilman RH Evans CA Optimizing tuberculosis testing for basic laboratories Am J Trop Med Hyg 2010 83 896 901 10.4269/ajtmh.2010.09-0566 20889887 PMC2946764

[B25] Daley P Michael JS S K Latha A Mathai D John KR Pai M A pilot study of short-duration sputum pre-treatment procedures for optimising smear microscopy for tuberculosis PLoS One 2009 4 e5626 10.1371/journal.pone.0005626 19461963 PMC2680966

